# Understanding of Authorship Guidelines and the Frequency of Authorship Misuse: A Descriptive Cross-Sectional Study in the State of Qatar

**DOI:** 10.1177/15562646251395350

**Published:** 2025-11-18

**Authors:** Seba Qussini, Samer Hammoudeh, Saad Shahbal, Rajvir Singh, Kris Dierickx

**Affiliations:** 1 Faculty of Medicine, Centre for biomedical ethics and law, 26657 KU Leuven, 3000 Leuven, Belgium; 2 The Medical Research Center, 532070 Hamad Medical Corporation, Doha, Qatar; 3 College of Health Sciences, 61780 Qatar University, Doha, Qatar; 4 Department of Medicine, 532070 Hamad Medical Corporation, Doha, Qatar; 5 36977 Heart Hospital, Hamad Medical Corporation, Doha, Qatar

**Keywords:** research integrity, honorary authorship, ghost authorship, ethics, Qatar, and authorship misuse

## Abstract

Ethical authorship practices ensure both accountability and credibility. In this study, we estimated the frequency of encountering honorary and ghost authorship at least once among researchers at Hamad Medical Corporation (HMC) in Qatar. Additionally, we evaluated researchers’ familiarity with standard authorship guidelines. Using a cross-sectional design, we administered a pre-developed anonymous online survey to 4043 researchers. Descriptive statistics in the form of percentages and frequences along with a 2-sided Chi-square tests were used. Significance was defined as p ≤ .05. Overall, researchers demonstrated low awareness of adopted authorship guidelines. While 24% of respondents reported never having heard of the International Committee of Medical Journal Editors (ICMJE) guidelines, 76% were aware of them but unfamiliar with the content. Additionally, the low awareness coincided with reported frequencies of having encountered honorary and ghost authorship at least once—70.5% and 45.5%, respectively. In conclusion, authorship misuse is a significant issue in Qatar, and appears to occur at levels consistent with those found in international surveys. It remains a delicate matter that can be approached by promoting awareness, educating researchers, and encouraging adherence to guidelines.

## Introduction

In today's “publish or perish” research setting, publications have become the dominant metric for performance evaluation and career progression, evolving into a form of research currency with significant social, financial and academic implications (Aliukonis et al., 2020; McNutt et al., 2018). This pressure is intensified by fierce career competition, coupled with depleted employment and funding opportunities (Aliukonis et al., 2020). These dynamics have contributed to a notable rise in publication counts and the emergence of hyperauthorship–a phenomenon where research articles are authored by hundreds to thousands of authors (Fire & Guestrin, 2019). These trends pose serious threats to research integrity, raising concerns about the nature of researchers’ authorship practices, suggesting potential misuse (Goddiksen et al., 2023). According to a systematic review, an estimated pooled weighted average of 29% of researchers experienced or reported others’ experiences with authorship misuse (Marušić et al., 2011).

The most prevalent forms of authorship misuse are honorary and ghost authorship (Resnik & Master, 2011). Honorary authorship refers to the practice of gifting unmerited authorship to a researcher who had no or minimal involvement in the research (Resnik & Master, 2011; Wislar et al., 2011). This could be out of several motives such as coercion, seniority, or gratitude (Aliukonis et al., 2020). On the other hand, ghost authorship is omitting a person's name as an author or co-author from a resulting publication despite his/her substantial involvement (Resnik & Master, 2011; Wislar et al., 2011). This may occur either with the individual's knowledge and consent (e.g., professional writers in industry-initiated trials) or without their consent (Gøtzsche et al., 2007). Authorship misuse is a growing concern as it could lead to serious implications, such as the loss of trust within the scientific community and between the society and scientists, diluting the contributions of true authors and intellectual property infringement (Aliukonis et al., 2020). Simultaneously, numerous efforts have been directed to enhance transparency in authorship, these efforts include developing authorship guidelines, such as the International Committee of Medical Journal Editors (ICMJE) criteria, the World Association of Medical Editors (WAME) (Patience et al., 2019), or the Committee on Publication Ethics (COPE) (Turner, 2024). In addition to authorship guidelines, other measures have been implemented, including the Contributor Roles Taxonomy (CRediT) methodology for contributions, defining authors’ roles and responsibilities (for journals) and the use of Open Researcher & Contributor ID (ORCID) identifiers (McNutt et al., 2018).

Despite the presence of multiple authorship guidelines, there remains considerable variation in authorship practices across disciplines and cultures (Hesselmann et al., 2021), and a lack of consensus on clear operational definition of authorship (Sheikh, 2000). Arguably, this could increase the risk of authorship misappropriation and misconduct (Schroter et al., 2020), and necessitates continuous efforts to understand researchers’ authorship practices and awareness of available guidelines. Funding agencies and research institutions have an important role to play in such endeavors, as tackling misconduct at the publication stage can often be recognized as too late, hence fostering good research practices at the early stages of research is both crucial and preventive (McNutt et al., 2018; Schroter et al., 2020). Authorship practices have been characterized and investigated in many countries (De Peuter et al., 2024; Goddiksen et al., 2023; Schroter et al., 2020); however, this topic has been under investigated in the Arab world (Alshogran & Al-Delaimy, 2018; Elgamri et al., 2023). To our knowledge, there are no previous studies that have explored researchers’ authorship practices in the State of Qatar. Given the existing gap in the literature, this study contributes to understanding authorship guidelines and misuse among researchers at the largest healthcare provider in the State of Qatar, Hamad Medical Corporation (HMC). The aim is to estimate the frequency of encountering authorship misuse at least once, specifically in the forms of honorary and ghost authorship, and to evaluate researchers’ awareness of authorship guidelines.

## Methods

### Tool

A web-based anonymous questionnaire was distributed to participants via an invitation email containing a link to the Microsoft Forms survey. The questionnaire consisted of 12 items and was developed and piloted by Schroter et al. (Schroter et al., 2020). There were also 6 additional demographic-related questions, and a free text box for additional comments (Appendix 1). The first four questions represent the respondent's familiarity with existing authorship guidelines internationally and within their institutions. Questions 5 and 6 aim to capture the frequency of encountering honorary authorship and ghost authorship, respectively. Notably, questions 5 and 6 included the definitions of honorary and ghost authorship in relation to the respondents’ experiences in research projects where gift or ghost authorship was encountered. Questions 7 - 12 assess current exhibited authorship practices and experiences with authorship misappropriation. Permission to use the tool was sought from Dr. Sara Schroter via email prior to project initiation. The tool was pretested on four participants, and minor revisions were implemented to adapt it to the HMC setting and to improve flow and clarity. The survey was administered in a single-page format, requiring respondents to provide answers to all questions, except for demographic section, which remained optional. The average time for completion was 13 min.

#### Study Setting

The study was conducted at the Medical Research Center (MRC), affiliated with Hamad Medical Corporation (HMC). HMC is the principal healthcare provider in the State of Qatar and comprises a network of specialized hospitals and institutions. The MRC functions as HMC's corporate research department, overseeing the receipt, review, funding, and management of research activities across the organization.

#### Participants

The recipients of the questionnaire were registered users on the institution's online research submission portal − ABHATH. These users were local and international researchers from various institutions who collaborated with HMC, and submitted research proposals at MRC, since July 2017, the launch date of the online submission portal. This included all team members: primary investigators, co-investigators, mentors, research coordinators and assistants. However, responses were only received from researchers within the organization; no external participants joined the study. Consequently, the final sample reflects researchers based in Qatar—both Qataris and non-Qataris—representing various biomedical disciplines.

#### Data Management

An email containing a link to the questionnaire was sent to all participants. It is important to note that data collection was carried out in English, which—alongside Arabic—is one of the official languages within the organization. Researchers are also typically requested to submit their research proposals in English.

The collected data were exported as an Excel file and subsequently uploaded into SPSS version 29 for processing and analysis. Data collection was conducted over June and July 2024, with three reminder emails sent at two-week intervals. No identifying information was collected; instead, serial codes were assigned to each dataset. All data were stored in a secure file accessible only to the study team.

#### Ethical Considerations

All study activities commenced post obtaining approval from the institutional review board (IRB) at Hamad Medical Corporation. The study was conducted in compliance with the Declaration of Helsinki Guidelines.

#### Consent to Participate

The requirement of a consent form was waived by the IRB. Alternatively, an information sheet was used and attached to the invitation email, detailing the purpose of the study, voluntary participation, and confidentiality measures. No financial incentives were offered in exchange for participation.

### Statistical Analysis

Statistical analysis was conducted utilizing SPSS version 29. A two tailed p value of ≤ 0.05 was considered for statistical significance. Demographic variables were presented as frequencies and valid percentages including variables related to ghost and honorary authorship. The frequency of encountering ghost and honorary authorship at least once was further stratified by age and number of publications. A chi-square test was used to evaluate the relationship between categorical variables.

## Results

A total of 333 responses were received (out of 4043 sent). This sample resembles Qatar-based biomedical researchers affiliated with HMC. It is noteworthy to mention that 343 emails were returned as undeliverable, therefore the response rate (9%) was calculated based on the remaining 3700 who received the email. The majority of respondents were in the 36–49 years age group (173, 52.6%) and had less than 5 years of active research experience (134, 41.1%), with 63% having published less than 10 articles in peer-reviewed journals as shown in Table 1.

**Table 1.** Characteristics of the Study Respondents.

	Response	N (%)
Age Category	21–35	53 (16.1)
	36–49	173 (52.6)
	50–65	95 (28.9)
	Above 65	8 (2.4)
Approximately, how many years have you been an active researcher?	Less than or equal to 5 years	134 (41.1)
	5 to 10 years	91 (27.9)
	More than 10 years	101 (31)
Approximately, how many papers have you published in a peer-reviewed journal as either an author or co-author?	Less than 10	201 (63)
	10 to 50	90 (28.2)
	More than 50	28 (8.8)
Institutions	HMC	333 (100)
Designation Category	Clinical	236 (81.9)
	Researcher	28 (9.7)
	Administration	19 (6.6)
	Others	5 (1.7)

Familiarity with the Adopted Authorship Criteria:

Table 2 below presents the responses received for questions related to familiarity with existing authorship guidelines and authorship practices and experiences. Regarding familiarity with the adopted authorship criteria, the majority of respondents (253, 76.2%) had heard of the ICMJE but were unfamiliar with the content. Nevertheless, most respondents (177, 53.7%) declared that the use of authorship guidelines is at least sometimes encouraged in their institution and have believed that explicit use of authorship criteria is beneficial in manuscript preparation (292, 88%). In practice, 43.6% reported having used authorship criteria explicitly to decide on authorship in their last published article. More details regarding respondents’ authorship practices and experiences are summarized in Table 2.

**Table 2.** Responses Received for Questions Related to Familiarity with Existing Authorship Guidelines and Authorship Practices and Experiences.

Question 1-8	Possible Answers	N (%)
Does your institution have authorship policy criteria researchers should use when deciding on who should be an author on a research paper?	Yes	210 (63.1)
	No	22 (6.6)
	I don’t know	100 (30)
	Not applicable	1 (0.3)
How familiar are you with the ICMJE criteria for authorship?	I have never heard of them	79 (23.8)
	I have heard of them, but I wasn’t familiar with the content	253 (76.2)
	I am very familiar with the content	0 (0)
In your current research setting, are the use of explicit authorship guidelines/criteria (for example ICMJE or institutional guidelines) actively encouraged?	Yes, they are frequently encouraged	85 (25.8)
	Yes, they are sometimes encouraged	92 (27.9)
	No, they are not encouraged	49 (14.8)
	I’m not sure	102 (30.9)
	Other	2 (0.6)
Do you think the explicit use of authorship guidelines/ criteria are beneficial to research teams when preparing/ writing a scientific paper and deciding on authorship?	Yes	292 (88)
	No	8 (2.4)
	I don’t know	32 (9.6)
Thinking of the last paper you coauthored, were explicit authorship criteria used to decide WHO should be an author?	Yes	142 (43.6)
	No	87 (26.7)
	I don’t know	97 (29.8)
Thinking of the last paper you coauthored, do you feel that the decision on WHO should be an author was a fair reflection of who did what?	Yes	191 (58.2)
	No	79 (24.1)
	I don’t know	58 (17.7)
Thinking of the last paper you coauthored, approximately how many times was authorship ORDER discussed by the research team?	Never	85 (25.9)
	Only once	78 (23.8)
	A few times	135 (41.2)
	Lots of times	30 (9.1)
Thinking of the last paper you coauthored, do you feel that the decision on the ORDER of authorship was a fair reflection of who did what?	Yes	190 (58.3)
	No	68 (20.9)
	I don’t know	68 (20.9)

Frequency of Encountering Authorship Misuse (Table 3):

**Table 3.** The Frequency of Encountering Ghost and Honorary Authorship at Least Once.

Question	Category	N (%)
How frequently have you been involved in a study where someone has been added as an author who did not contribute substantially to the conception or the design of the work, or the acquisition, analysis, or interpretation of data for the work; or the writing of the article?	Never	98 (29.5)
	Once	41 (12.4)
	A few times	138 (41.7)
	Lots of times	54 (16.3)
How frequently have you been involved in a study where someone was not listed as an author when they contributed substantially to the conception or the design of the work, or the acquisition, analysis, or interpretation of data for the work; or the writing of the article?	Never	181 (54.5)
	Once	45 (13.6)
	A few times	91 (27.4)
	Lots of times	15 (4.5)

Gift authorship was encountered more frequently than ghost authorship. A total of 70.5% of the respondents reported encountering at least once gift authorship, whereas ghost authorship was encountered at least once by 45.5% as shown in Table 3. Given that the majority of respondents were clinically affiliated (81.9%), stratification by discipline revealed that the frequency of encountering honorary and ghost authorship at least once, was predominantly reported by clinicians (Supplementary File 1).

Table 4 presents the frequency of encountering ghost authorship stratified by age group and number of publications. While no statistically significant differences were observed, some notable trends emerged. Mid-career researchers (36–49) consistently reported the highest percentages of encountering ghost authorship, particularly at higher publication counts. Among researchers with less than 10 publications, the highest percentage for encountering ghost authorship a few times (62.1%) was by mid-career researchers.

**Table 4.** Frequency of Encountering Ghost Authorship by age Category and Publications.

		Never n (%)	Once n (%)	Few times n (%)	Many times n (%)	P-value
Less than 10	21–35	26 (25.2)	5 (17.9)	8 (13.8)	4 (33.3)	0.808
	36–49	53 (51.5)	17 (60.7)	36 (62.1)	6 (50)	
	50–65	24 (23.3)	6 (21.4)	14 (24.1)	2 (16.7)	
	Above 65	0	0	0	0	
10 to 50	21–35	3 (5.8)	1 (10)	4 (15.4)	0	0.467
	36–49	25 (48.1)	5 (5)	11 (42.3)	1 (100)	
	50–65	22 (42.3)	3 (30)	10 (38.5)	0	
	Above 65	2 (3.8)	1 (10)	1 (3.8)	0	
More than 50	21–35	1 (6.3)	0	0	0	0.334
	36–49	5 (31.3)	4 (80)	1 (20)	2 (100)	
	50–65	9 (56.3)	0	2 (40)	0	
	Above 65	1 (6.3)	1 (20)	2 (40)	0	

In a similar fashion, Table 5 shows the frequency of encountering honorary authorship stratified by age group and number of publications. Likewise, mid-career researchers (36–49) generally reported the highest frequencies for encountering honorary authorship at least once. Additionally, for encountering honorary authorship multiple times, older researchers (>50 years old), are less likely to report encountering honorary authorship.

**Table 5.** Frequency of Encountering Honorary Authorship by age Category and Publications.

		Never n (%)	Once n (%)	Few times n (%)	Many times n (%)	P-value
Less than 10	21–35	11 (18)	7 (23.3)	16 (19.5)	8 (29.6)	0.81
	36–49	36 (59)	18 (60)	44 (53.7)	14 (51.9)	
	50–65	14 (23)	5 (16.7)	22 (26.8)	5 (18.5)	
	Above 65	0	0	0	0	
10 to 50	21–35	1 (4.2)	1 (11.1)	2 (5.1)	4 (23.5)	0.47
	36–49	13 (54.2)	3 (33.3)	19 (48.7)	7 (41.2)	
	50–65	10 (41.7)	4 (44.4)	16 (41)	5 (29.4)	
	Above 65	0	1 (11.1)	2 (5.1)	1 (5.9)	
More than 50	21–35	0		0	1 (10)	0.33
	36–49	1 (16.7)		5 (41.7)	6 (60)	
	50–65	3 (50)		5 (41.7)	3 (30)	
	Above 65	2 (33.3)		2 (16.7)	0	

**Figure 1:**
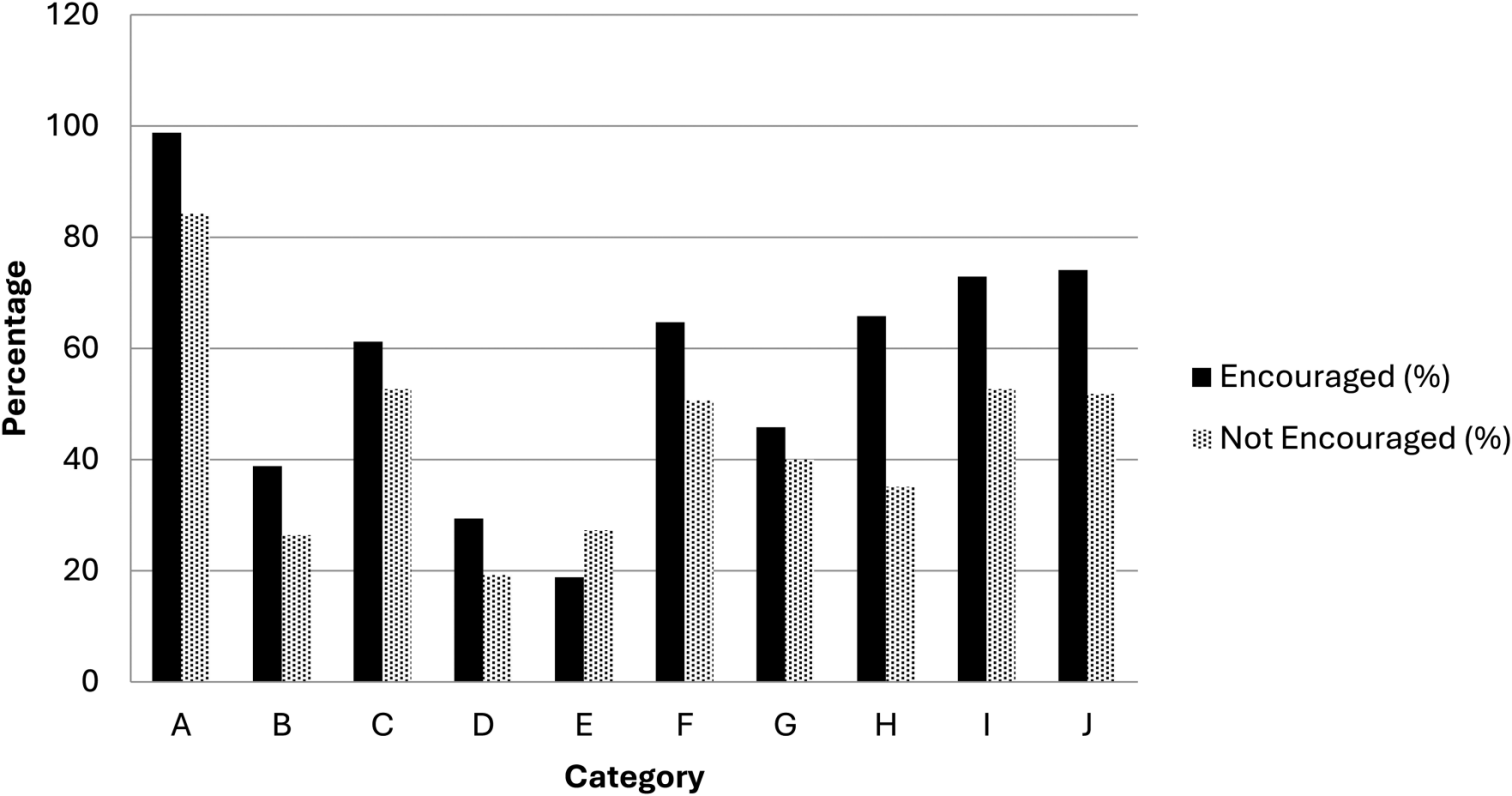
Stratification of Responses by Whether the use of Explicit Authorship Guidelines and Criteria in Current Research Institution/Department is Frequently Encouraged or not.The bars represent the following statements: (A) Agrees That the Explicit use of Authorship Guidelines and Criteria is Beneficial to Research Teams When Preparing a Paper and Deciding on Authorship, (B) Never Been Involved in a Study Where Someone has Been Added as an Author who did not Contribute Substantially (Honorary Authorship), (C) Never Been Involved in a Study Where Someone was not Listed as an Author When They Contributed Substantially (Ghost Authorship), (D) Never Experienced Honorary or Ghost Authorship, (E) Experienced Both Honorary and Ghost Authorships, (F) Authorship Eligibility Discussed at an Early Stage During Study Design, (G) Authorship Order Discussed at an Early Stage During Study Design, (H) Used Explicit Authorship Criteria to Decide who Should be an Author on Their Last Coauthored Paper, (I) Felt Decision on who Should be an Author on Their Last Coauthored Paper was a Fair Reflection of who did What, (J) Felt Decision on Order of Authorship on Their Last Coauthored Paper was a Fair Reflection of who did What.

Encouraging the Use of Authorship Guidelines:

Figure 1 reflects the effects of departmental practices encouraging the frequent use of authorship guidelines. Collectively, respondents who reported frequent encouragement were more likely to indicate that authorship decisions were discussed at earlier stages and were perceived as fairer reflections of merit-based contributions. Moreover, these respondents were less likely to report encountering incidents of honorary or ghost authorship compared with those who did not experience such encouragement.

## Discussion

This study aimed to depict the scope of authorship misuse among researchers at Hamad Medical Corporation, the largest healthcare provider in the State of Qatar. Additionally, we evaluated researchers’ familiarity with standard authorship guidelines (International Committee of Medical Journal Editors, 2025), and the potential effects of frequently encouraging authorship use. Using a descriptive cross-sectional design, this study estimated the frequency of encountering (at least once) honorary authorship at 70.5% and ghost authorship at 45.5%. Regarding familiarity with the adopted authorship criteria, researchers demonstrated low awareness, as 24% of respondents reported never having heard of the ICMJE guidelines, while 76% had heard of them but were unfamiliar with their content.

Although this study represents only a preliminary step in characterizing authorship practices among researchers at HMC in the State of Qatar, the estimated frequency of encountering of authorship misuse is of concern. A recent systematic review reported a pooled prevalence of 26% researchers who experienced honorary authorship in settings lacking clear authorship guidelines (Meursinge Reynders et al., 2024). Similarly, Schroter and colleagues investigated authorship misuse utilizing the same tool employed in this study, reported a frequency of 74% and 34% for encountering honorary and ghost authorships, respectively (Schroter et al., 2020).

The frequencies of encountering authorship misuse estimated in this study could be attributed to several factors. First, it may stem from researchers’ lack of awareness of the ICMJE criteria (Claxton, 2005; Schroter et al., 2020). In this context, HMC has established authorship criteria in their published policy regarding this matter, which also includes arrangements for authorship disputes. However, limited familiarity with established authorship guidelines can make it more challenging for researchers to define authorship in a unified manner, increasing the likelihood of misappropriation (Alshogran & Al-Delaimy, 2018). Second, early-career researchers are likely to exhibit limited familiarity with authorship practices, which may lead to higher rates of authorship misuse (Alshogran & Al-Delaimy, 2018). This factor is particularly relevant, as researchers with less than five years of experience constituted 41% of our total sample. Third, the current “publish or perish” culture in today's research landscape, coupled with the reliance on flawed proxy measures, such as publication counts to evaluate research performance or for promotion, has disrupted the research ecosystem (Edwards & Roy, 2016). This has led to unrealistic productivity expectations (Macleod et al., 2014) and an indirect endorsement of questionable research practices (Kamerlin, 2015). Additionally, some institutional dynamics, such as hierarchal relationships, reciprocated favors, or nepotism, may further contribute to researchers’ engagement in authorship misuse (Schroter et al., 2020; Tsai et al., 2016). As such, it is also recommended that institutions adopt practices and policies that promote ethical authorship practices and address negative cultural factors. For example, the Declaration on Research Assessment (DORA) offers robust recommendations for research evaluation, emphasizing the responsible use of metrics in decisions related to promotion, funding, and assessment (Declaration on Research Assessment, 2012). Moreover, the 5 principles of The Hong Kong Principles for Assessing Researcher aim to promote research integrity by shifting the focus from traditional metrics (such as the impact factor and H-Index) to researchers’ behaviours that can foster responsible, trustworthy research (Moher et al., 2020).

When compared with other disciplines, authorship malpractices in psychology journals show comparable rates, with 64.1% for gift authorship and 26.6% for ghost authorship (De Peuter et al., 2024). In journals within the business domain, 44.8% of respondents reported collaborating on a research article where authors were added despite making little to no contributions (Manton & English, 2008). Similarly, an investigation in the social sciences revealed that 43.38% of published articles had at least one added author with no contribution to the work (Pruschak & Hopp, 2022). Collectively, these findings might suggest that this is far from being a discipline-specific concern, but rather an interdisciplinary issue requiring joint efforts.

While our results provide an estimate of authorship misuse among researchers at HMC in Qatar and suggest a potential link between career stage, publication productivity, and exposure to authorship misuse; interpreting the findings comes with challenges. The reported frequencies for encountering authorship misuse at least once could be due to limited familiarity with established authorship guidelines amongst junior researchers, as mentioned earlier. Alternatively, it may result from their exposure to such practices by senior colleagues. This dynamic could also explain the low representation of senior researchers in our sample, possibly due to reluctance to participate linked to their own involvement in authorship misuse—a phenomenon commonly referred to as self-selection bias (Ibbett et al., 2023). Moreover, interpreting the frequency of encountered authorship misuse presents challenges, as multiple respondents may have reported the same incident, thereby inflating the results (De Peuter et al., 2024).

## Educational Implications

The findings of this study, particularly the low familiarity with the adopted guidelines, shed light on the urgent need for informed educational modules on responsible authorship practices. As such, institutions are urged to explore the development and implementation of training modules on research ethics to strengthen researchers’ intrinsic sense of research integrity. Raising awareness of the importance of ethical authorship practices and providing clear instructions and definitions on the adopted criteria might help reduce authorship misappropriation.

## Research Agenda

This study explored a critical area of research integrity that is seldom discussed. Such efforts are needed not only in the State of Qatar but also in the region, where the body of evidence in this domain remains scarce (Alshogran & Al-Delaimy, 2018; Elgamri et al., 2023). Analytical studies should promptly follow to further investigate underlying factors and establish associations. Future research could explore the impact of authorship awareness campaigns and educational modules on reducing authorship misuse and investigate authorship practices among senior researchers, or comparing these between academic and non-academic institutions. Finally, seniority as a motive for authorship misappropriation should be further investigated and managed appropriately.

## Best Practices

Research institutions and journals are encouraged to review and revise their internal processes and authorship policies to align with more versatile and universal definitions, such as the McNutt definition, where she defines merited authorship as contributing to either conception or design, acquisition, analysis, or interpretation of data, creation of new software, or drafting or revising the manuscript—along with agreeing to accountability and final approval of the version (McNutt et al., 2018). Many distinguished journals, such as Nature, have adopted the more inclusive McNutt et al. definition (*Nature Portfolio*, n.d*.*). Moreover, as mentioned above, institutions can adopt policies that counter, or at least minimize, authorship malpractices. By shifting the focus towards researchers’ behaviors and other scholarly outputs—such as peer review activities, datasets, or mentorship—healthy competition can be maintained, and the aimless race to publish more could ultimately be curbed (Moher et al., 2020). Institutions and universities are also encouraged to adopt training modules that promote the responsible conduct of research. Recent evidence supports the positive outcome of such training on researchers’ confidence and knowledge in recognizing and avoiding research misconduct (Lake-Bullock et al., 2024), particularly in areas such as authorship (Plemmons et al., 2006).

## Limitations

Although we aimed to provide a preliminary characterization of authorship misuse among researchers at HMC in Qatar, we acknowledge several limitations that may have influenced—or potentially inflated—the reported frequencies. Primarily, these limitations include the low response rate which might have introduced nonresponse bias (Berg, 2005). However, the current response rate is comparable to previous experiences in the same setting (AlMulla et al., 2021; Elewa et al., 2015; Khoodoruth et al., 2021; Qussini et al., 2024) and falls within the acceptable range for online surveys (Wu et al., 2022). Second, the use of a non-validated tool may have resulted in nuanced understandings of the questions and varied interpretations by respondents. Notably, the tool adopts a broad definition for ghost authorship, capturing both consensual omission (e.g., professional writers) and non-consensual unjust exclusion. This may have contributed to misinterpretation among respondents. However, the tool has been previously piloted, published, and utilized in other studies (De Peuter et al., 2024; Schroter et al., 2020). Third, relying on self-reported data could have led to overreporting instances of authorship misuse (Bauhoff, 2023). Moreover, as the adopted tool inquired about instances of encountered authorship malpractices, previous research suggests that reported rates of such instances could be higher than those of self-admitted misconduct (Godecharle et al., 2018). Finally, although HMC researchers constitute a significant portion of researchers in Qatar, our sample might not fully represent the broader population of researchers in the state of Qatar. This limitation arises from the fact that received responses were exclusively from researchers registered on the online submission portal.

## Supplemental Material

sj-docx-1-jre-10.1177_15562646251395350 - Supplemental material for Understanding of Authorship Guidelines and the Frequency of Authorship Misuse: A Descriptive Cross-Sectional Study in the State of Qatar Supplemental material, sj-docx-1-jre-10.1177_15562646251395350 for Understanding of Authorship Guidelines and the Frequency of Authorship Misuse: A Descriptive Cross-Sectional Study in the State of Qatar by Seba Qussini, Samer Hammoudeh, Saad Shahbal, Rajvir Singh and Kris Dierickx in Journal of Empirical Research on Human Research Ethics

sj-docx-2-jre-10.1177_15562646251395350 - Supplemental material for Understanding of Authorship Guidelines and the Frequency of Authorship Misuse: A Descriptive Cross-Sectional Study in the State of Qatar Supplemental material, sj-docx-2-jre-10.1177_15562646251395350 for Understanding of Authorship Guidelines and the Frequency of Authorship Misuse: A Descriptive Cross-Sectional Study in the State of Qatar by Seba Qussini, Samer Hammoudeh, Saad Shahbal, Rajvir Singh and Kris Dierickx in Journal of Empirical Research on Human Research Ethics

## Abbreviations

HMC: Hamad Medical Corporation
CRediT: Contributor Roles Taxonomy
ORCID: Open Researcher & Contributor ID
ICMJE: International Committee of Medical Journal Editors
WAME: World Association of Medical Editors
COPE: Committee on Publication Ethics
MRC: Medical Research Center
IRB: Institutional Review Board
DORA: Declaration on Research Assessment
