# Supplementary material for: Understanding of Authorship Guidelines and the Frequency of Authorship Misuse: A Descriptive Cross-Sectional Study in the State of Qatar
Source: J Empir Res Hum Res Ethics. 2025 Nov 18;21(1-2):39–48. doi: 10.1177/15562646251395350 (PMC12913682; doi:10.1177/15562646251395350)
Supplement: sj-docx-1-jre-10.1177_15562646251395350 - Supplemental material for Understanding of Authorship Guidelines and the Frequency of Authorship Misuse: A Descriptive Cross-Sectional Study in the State of Qatar [file sj-docx-1-jre-10.1177_15562646251395350.docx]

**Supplementary File: Frequencies of Observing Ghost/ Honorary Authorship Stratified by Designation Category**

| **Ghost Authorship** |  | **Clinical**  **n (%)** | **Research**  **n (%)** | **Admin/ Others**  **n (%)** | **P-value** |
| --- | --- | --- | --- | --- | --- |
|  | Never  N=85 (29.6) | 67 (78.8) | 7 (8.2) | 11 (13) | 0.17 |
|  | Once  N=37 (12.9) | 32 (86.5) | 1 (2.7) | 4 (10.8) |  |
|  | Few Times  N=120 (41.8) | 102 (85) | 13 (10.8) | 5 (4.1) |  |
|  | Many Times  N=45 (15.7) | 34 (75.6) | 7 (15.6) | 4 (8.8) |  |
| **Honorary Authorship** | Never  N=161 (55.9) | 129 (80.1) | 16 (9.9) | 16 (10) | 0.001 |
|  | Once  N=39 (13.5) | 33 (84.6) | 3 (7.7) | 3 (7.7) |  |
|  | Few Times  N=78 (27.1) | 69 (88.5) | 6 (7.7) | 3 (3.8) |  |
|  | Many Times  N=10 (3.5) | 5 (50) | 3 (30) | 2 (20) |  |
