# Supplementary material for: Understanding of Authorship Guidelines and the Frequency of Authorship Misuse: A Descriptive Cross-Sectional Study in the State of Qatar
Source: J Empir Res Hum Res Ethics. 2025 Nov 18;21(1-2):39–48. doi: 10.1177/15562646251395350 (PMC12913682; doi:10.1177/15562646251395350)
Supplement: sj-docx-2-jre-10.1177_15562646251395350 - Supplemental material for Understanding of Authorship Guidelines and the Frequency of Authorship Misuse: A Descriptive Cross-Sectional Study in the State of Qatar [file sj-docx-2-jre-10.1177_15562646251395350.docx]

***Appendix 1 (Authorship Guidelines and Usage Survey):***

1. Does your institution/main work location have authorship policy criteria researchers should use when deciding on who should be an author on a research paper?

- Yes
- No
- I don’t know
- Not applicable

ICMJE Criteria for Authorship

The international Committee of Medical Journals Editors (ICMJE) recommends that authors be based on the following 4 criteria:

- Substantial contributions to the conception or design of the work; or the acquisition, analysis, or interpretation of data for the work; AND
- Drafting the work or reviewing it critically for important intellectual content; AND
- Final approval of the version to be published; AND
- Agreement to be accountable for all aspects of the work in ensuring that questions related to the accuracy or integrity of any part of the work are appropriately investigated and resolved.

1. How familiar are you with the ICMJE criteria for authorship listed above>

- I have never heard of them
- I have heard of them, but I wasn’t familiar with the content
- I am very familiar with the content

1. In your current research setting, are the use of explicit authorship guidelines/criteria (for example ICMJE or institutional guidelines) actively encouraged?

- Yes, they are frequently encouraged
- Yes, they are sometimes encouraged
- No, they are not encouraged
- I’m not sure
- Other (please specify):

1. Do you think the explicit use of authorship guidelines/criteria are beneficial to research teams when preparing/writing a scientific paper and deciding on authorship?

- Yes
- No
- I don’t know

1. How frequently have you been involved in a study where someone has been added as an author who did not contribute substantially to the conception or the design of the work, or the acquisition, analysis, or interpretation of data for the work; or the writing of the article?

- Never
- Once
- A few times
- Lots of times

1. How frequently have you been involved in a study where someone was not listed as an author when they contributed substantially to the conception or the design of the work, or the acquisition, analysis, or interpretation of data for the work; or the writing of the article?

- Never
- Once
- A few times
- Lots of times

1. Thinking of the last paper you coauthored, at what point in time were details about WHO should be an author discussed? [Tick all that apply]

- At an early stage during the design of the study
- During the course of the study
- Once the study was completed and before writing the paper
- During paper writing
- After the paper was written
- It was never discussed

1. Thinking of the last paper you coauthored, at what point in time were details about THE ORDER of authorship discussed? [Tick all that apply]

- At an early stage during the design of the study
- During the course of the study
- Once the study was completed and before writing the paper
- During paper writing
- After the paper was written
- It was never discussed

1. Thinking of the last paper you coauthored, were explicit authorship criteria used to decide WHO should be an author?

- Yes
- No
- I don’t know

1. Thinking of the last paper you coauthored, do you feel that the decision on WHO should be an author was a fair reflection of who did what?

- Yes
- No
- I don’t know

1. Thinking of the last paper you coauthored, approximately how many times was authorship ORDER discussed by the research team?

- Never
- Only once
- A few times
- Lots of times

1. Thinking of the last paper you coauthored, do you feel that the decision on the ORDER of authorship was a fair reflection of who did what?

- Yes
- No
- I don’t know

And finally, some questions about yourself:

1. For which department and institution do you mainly work for?
2. What is your current designation?
3. What is your age?
4. Approximately, how many years have you been an active researcher?
5. Approximately, how many papers have you published in a peer-reviewed journal as either an author or co-author?
6. Do you have further comments?

Thank you for your help with this research. Please now click "submit" to complete the survey.
